# Short and long-term career plans of final year dental students in the United Arab Emirates

**DOI:** 10.1186/1472-6831-13-39

**Published:** 2013-08-13

**Authors:** Hazim H Rashid, Swapnil G Ghotane, Salem H Abufanas, Jennifer E Gallagher

**Affiliations:** 1 Al Dhaid Hospital, Ministry of Health, Sharjah, United Arab Emirates; 2 King’s College London Dental Institute at Guy’s, King’s College and St Thomas’s Hospitals Dental Public Health, London, UK; 3 College of Dentistry, Ajman University of Science and Technology, Ajman, United Arab Emirates

**Keywords:** Dental student, Career expectations, Dentistry, United Arab Emirates

## Abstract

**Background:**

New dental schools have been established to train dentists in many parts of the world. This study examines the future dental workforce from the first dental school in the United Arab Emirates [UAE]; the aim of this study was to explore the short and long-term career aspirations of the final year dental students in the UAE in relation to their demography.

**Method:**

Final year dental students of the Ajman University’s College of Dentistry (n=87) were invited to participate in a self-completion questionnaire survey. Descriptive analysis, chi-square tests, and binary logistic regression analysis were carried out on career aspirations using SPSS v20.

**Results:**

Eighty-two percent of students (n=71) responded, the majority of whom were female (65%; n=46). Ethnicity was reported as: ‘other Arab’ (61%; n=43), ‘Emirati’ (17%, n=12), and ‘Other’ (21%, n=15). In the short-term, 41% (n=29) expressed a desire to work in government training centres, with Emirati students significantly more likely to do so (*p*=0.002). ‘Financial stability’ (80%; n=57) and ‘gaining professional experience’ (76%; n=54) emerged as the most important influences on their short-term career plans. The vast majority of students wished to specialise in dentistry (92%; n=65) in the longer term; logistic regression analysis revealed that the odds of specialising in the most popular specialties of Orthodontics and Oral and Maxillofacial Surgery were less for the ‘Other’ ethnic group when compared with ‘Emirati’ students (0.26; 95% CI 0.068-0.989; p=0.04). Almost three-quarters of the students overall (72%; n=51) intended to work full-time. ‘High income/financial security’ (97%; n=69), ‘standard of living’ (97%; n=69), ‘work/life balance’ (94%; n=67), and ‘professional fulfilment’ (87%; n=62) were reported by the students as the most influential items affecting their long-term professional career choices.

**Conclusion:**

The findings suggest that students aspire to make a long-term contribution to the profession and there is a high level of interest in specialisation with a desire to achieve financial stability and quality of life.

## Background

The healthcare workforce is an important element of society and a vital resource within healthcare [1], with dentistry being no exception [2]. This essential sector of the healthcare workforce is influenced by the wider context including political, societal, and economic change [3]. Gallagher *et al*., highlighted potential tensions between student motivation for selecting their professional care and the practice of dentistry; thus, it becomes imperative to understand the opinions and expectations of the prospective individuals to retain a motivated workforce [4].

UAE has an ethnically diverse population of around five million people [5]. In 1997, the College of Dentistry at Ajman University of Science & Technology was the first dental school to be established in the UAE and it is the only institute which has both undergraduate and post-graduate programs [6]. The curriculum of College of Dentistry consists of teaching and learning methods with a strong emphasis on critical problem solving and problem based learning using the multimedia and internet as learning information resources, together with practical simulation and clinical training [7].

Since the foundation of this dental school, five more institutions have been established which are accredited by the Commission of Academic Accreditation in the UAE [8]. Out of the five institutes, two have graduate and master’s courses only, whereas the other three institutes have only undergraduate dentistry programs [9-13]. Prior to 1997, there were no dental schools in the UAE and at that time there were about 777 dentists, all of whom had qualified abroad [14]. The development of dental training institutions, together with an influx of foreign qualified dentists, has led to a significant rise in the number of dentists. Over the last decade, the numbers almost quadrupled from 777 in 2000 to 2,916 in 2010 [15]. This increase has resulted in a competition for job opportunities for the new graduates. Moreover, limited availability of post-graduate training centres is an important concern for the graduates who wish to pursue further specialization in dentistry within UAE. In light of these issues, it is important to understand their career motivations and expectations so that, where possible, they may be harnessed in the provision of healthcare [16,17].

Previous qualitative and quantitative studies in the UK have revealed that the final year dental students aspire to attain ‘professional experience’, ‘independence’, and financial stability’ on a short-term basis whereas long-term goals involved improving ‘standard of living’, ‘balance between work and life’, and ‘achieving financial security’, largely within their country of training [2,4]. To date, there has been no information on the career expectations of dental students in the UAE.

### Objective of this study

The objective of this study was to investigate the short and long-term career aspirations of the final year dental students in the UAE with respect to their demographic status (gender and ethnicity).

## Method

Final year dental students (n=87) in the College of Dentistry at the Ajman University of Science & Technology (AUST) from the academic year 2006–07 were invited to participate in this study. Ethical approval was obtained from the research ethics committee of the dental school (Ref: Dental/7095/2007). A questionnaire developed for dental students by Gallagher *et al.*[3,18,19] was modified to ensure it was sensitive to local career options and piloted on a sample of fourth year dental students in order to check the length, clarity and ambiguity of the questions.

Students were invited by letter to take part in the survey and received an information sheet with details of the research. During an academic teaching session, the study was explained to students and they were given the chance to ask questions. Questionnaires were then distributed and the students were given the time and opportunity to complete and return the questionnaires, if they wished to do so. The students did not receive incentives to participate in the study and they were under no obligation to complete the questionnaire. They were informed that their completion of the questionnaire would imply their consent for this research and subsequent publication of the data.

The questionnaire included five main sections as follows: motivation for choice of professional career, short-term career options and influences, long-term career options and influences, views on state healthcare, and finally the personal demographic details of respondents. The instrument consisted of both closed and open-ended questions. A five point ‘Likert scale’ was used to score students’ views ranging from ‘very important’ (score ‘1’) to ‘not important at all’ (score ‘5’) in the responses.

The questionnaire section on short-term career aspirations investigated students’ preference for internship training centre and whether they intend to travel abroad after their studies. This section also explored students’ choice of ‘area of work’ on a short-term basis followed by questions on items influencing their professional career and professional goals they intend to achieve in the next five years. Likewise, the questions on long-term career expectations investigated features such as the ‘type of role’ they wish to play within dentistry, their ‘area of interest’, and the ‘type of setting’ in which they want to work. This section concluded with questions on their expectation about working hours and the items that they perceived might affect their working hours.

Descriptive and univariate analysis demonstrated an outline of findings of the study and sample characteristics. Chi-square tests were used to examine the association between different ethnic groups and their choice of training centre preference. The ‘Z’ test for comparing two proportions was used to test the significant difference between the ‘Emirati’ and ‘other Arab’ ethnic groups in selection of training centre. Binary logistic regression was carried out using ‘preference for specialty’ as the dependent variable, to explore the difference in selection of a specialty by means of ethnicity, sex and age. For all the tests, significance was adopted if the p value was less than 0.05 (5% level of significance), and all the analyses were carried out using SPSS version 20.0.

## Results

### Respondents’ characteristics

Out of the total population of 87 students, 71 participated in the study giving a response rate of 82%. The age of the students ranged from 21 to 29 years, with a mean of 23.8 and mode of 23 years. The proportion of females candidates (65%; n=46) was almost double that of males (35%; n=25). The distribution of students according to ethnicity was uneven with the majority reporting that they were ‘other Arab’ 61% (n=43) and 17% Emirati (n=12); Persian and Indian students were combined to form an ‘Other’ ethnic group (21%, n=15).

### Short-term career aspirations

#### Following graduation

**Figure 1:**
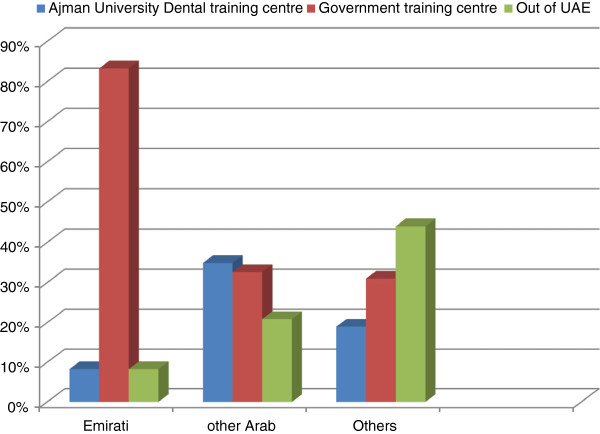
**Training centre preference among final year dental students by ethnicity (n = 71).** This figure reports the percentage of students (by ethnicity) preferring each of the three training centres namely Ajman University Dental training centre, government training centre, and the training centres based ‘out of UAE’.

The highest level of interest was to work in government training centres (41%; n=29), followed by Ajman University’s dental training centre (27%; n=19). This compared with 24% (n=17) who indicated a desire to undertake their internship training outside of UAE. The vast majority of Emirati (83%) students selected the government training centre against 33% of ‘other Arab’ students (Z=3.1, *p*=0.002) (Figure 1). The students were asked to justify their selection of training centre and among those who selected government training centres, 58% (n=17) stated that it provided ‘professional experience’ and 25% (n=7) gave ‘salaried training’ as the main reason for their selection. The majority of Persian students favoured taking their internship training outside of UAE (63.6%; n=7), because it involved the chance to ‘return back to home’.

#### Choice of work place

Students were asked about the items they perceived would influence their choice of work place in the short-term. ‘Job availability’ (92%; n=65) and ‘career opportunities’ (90%; n=64) emerged as the most important items influencing students’ decisions regarding their choice of workplace. Items such as ‘proximity to friends’ (37%; n=26), and working in a ‘rural area’ (24%; n=17), were the least important items.

#### Items influencing professional career in the short term

**Figure 2:**
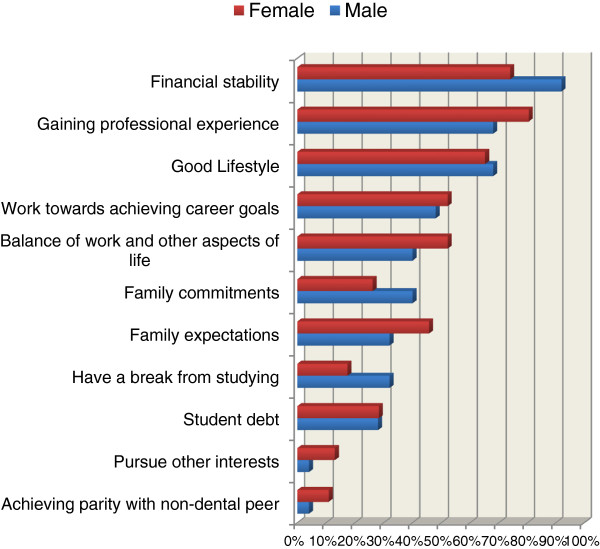
**Items perceived as influencing short-term professional career aspirations of final year dental students by gender (n = 71).** This figure reports the percentage of the items which students perceived as influencing their short term career expectations. Males and females are compared. The students could tick one or more items.

The majority considered ‘financial stability’ as the most important influence in decision making about their career in the short term (80%; n=57), particularly males (92%; n=23) (Figure 2). ‘Gaining professional experience’, was the second most important influence for students (76%; n=54), particularly amongst females (80%; n=37). Neither difference was statistically significant.

### Long-term career aspirations

#### Type of role and setting in dentistry

**Figure 3:**
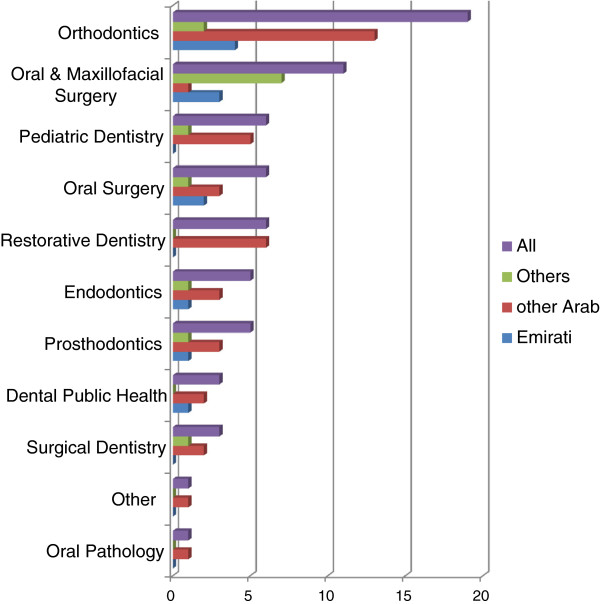
**Final year dental students preference for specialisation by specialty and ethnic group (n = 71).** This figure reports the percentage of dental specialities most preferred by the students. Ethnic groups are compared.

Participants were asked about the type of roles and setting in which they anticipated working in dentistry. The vast majority reported that they intended to specialise (92%, n=65). Orthodontics was the most favoured option followed by Oral and Maxillofacial Surgery (Figure 3). Logistic regression analysis revealed that the preference for Orthodontics and Oral and Maxillofacial Surgery (combined together) was significantly less for ‘Other’ (Persian/Indians) ethnic group as compared to ‘Emirati’ the reference group (Table 1); however, the preference for these specialities did not differ significantly between male and female students. Similarly, no significant difference was observed by age (p>0.05).

**Table 1:** Results of logistic regression analysis in finding the predictors of students’ preference for the specialties of orthodontics and oral and maxillofacial surgery (n=71)

**Predictors**	**Odds Ratio (OR)**	**95% C.L for OR**		**P value**
		**Lower C.L**	**Upper C.L**	
Sex	0.513	0.164	1.603	0.25
Age	0.793	0.556	1.133	0.20
*Ethnicity	-	-	-	0.08
**Other Arab**	0.710	0.138	3.642	0.68
**Others**	0.260	0.068	0.989	0.04

Note: * The ethnic group of ‘Emirati’ is selected as a reference point.

This table reports the odds ratio, by ethnicity, for students’ preference for specialty in dentistry. The ethnic group of ‘Emirati’ is selected as a reference point.

#### Work pattern

**Figure 4:**
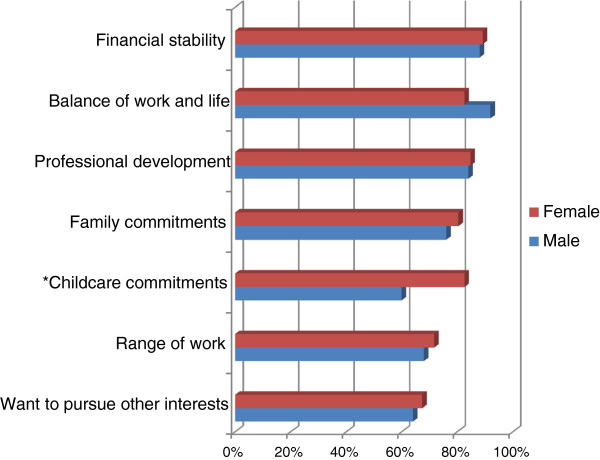
**Items perceived as important and very important influences on the number of working sessions per week of final year dental students, by gender (n = 71).** This figure reports the proportion of important and very important influences on the number of sessions they planned to work in the long term.

The survey explored the views of the students regarding their plans to work on a full-time or part-time basis in dentistry. Seventy-two per cent of students (n=51) planned to work full-time against 20% (n=14) part-time. Although males were more likely than females to report working full-time (84% cf 65%), this difference was not significant. The top items which emerged as an influence for their working pattern were ‘financial stability’ (89%; n=63),’balance of work and life’ (86%; n=61), and ‘professional development’ (84%; n=60) (Figure 4). ‘Childcare commitments’, was significantly more likely to be perceived as an issue by females (83%; n=38) compared with males (60%; n=15) (p=0.03).

#### Items influencing professional career in the long term

**Figure 5:**
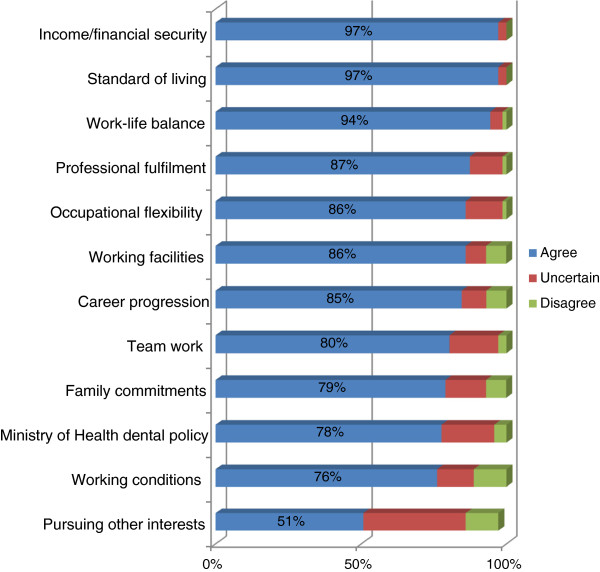
**Items perceived as influencing the long-term professional career aspirations of final year dental students (n = 71).** This figure gives the percentage of items which influences the long term career plans of the dental students. The responses for ‘strongly agree’ and ‘agree’ were grouped together and are presented under the response of ‘agree’. Similarly, responses for ‘strongly disagree’ and ‘disagree’ were grouped together and are presented as ‘disagree’.

All the students were asked to rate the factors that they believed would influence their future professional career. After combining ‘agree’ and ‘strongly agree’ influences, the vast majority of students concurred that both ‘income and financial security’ (97%; n=69) and ‘standard of living’ (97%; n=69) were the most important influences (Figure 5).

## Discussion

The findings of this paper provide insight into the career aspirations of senior dental students from one dental school, prior to their emerging into the dental profession. Three-quarters of the students wished to work in the UAE in the short-term, particularly in government training centres and the Ajman University’s Dental Training Centre; however, some ethnic groups wished to return home for their first position. Short-term goals were focused on ‘financial stability’ and ‘gaining professional experience’ which emerged as the most important influences. Long-term career goals and aspirations were very strongly orientated towards specialisation, particularly amongst Emiratis. The majority perceived that they would have a full-time commitment to dentistry. Student responses suggest that their future career plans are being influenced by financial, personal, and professional goals.

This study is part of a research programme to build greater understanding of the global dental workforce. The motivation of dental students in the UAE for selecting dentistry as a profession has already been reported [20].

### Short-term career aspirations

The main short-term goal for this cohort of students was to achieve ‘financial stability’. This may be explained by the fact that the College of Dentistry at Ajman University of Science & Technology is a private dental school, where majority of students are responsible for meeting their own fees. The students and their families, therefore carry a significant financial burden.

Furthermore, in the UAE a dental graduate has two options for post-qualification training: first, through government health institutes and second, through authorized private centres. Dental graduates have to pay for further training in the private centres, which is an additional financial burden. Thus, it is not surprising that these students aspire to achieve ‘financial stability’. Similar aspirations were found by Gallagher *et al*. [2] in the UK where dental education has historically been largely state funded. It is in line with sociologists’ analysis of professions which set out to achieve financial status as a key outcome of achieving professional status [21].

The second most important factor influencing the students’ short-term career expectations was ‘gaining professional experience’. More than half of the students who expressed their desire to train in government centres justified their selection relating to gaining professional experience. It is important for each of the new graduates to increase their skills and dexterity, assess their potential specialist interest and develop their independence after graduation; however, this is a potential concern for most new professionals and this need to gain professional experience was also reflected in the UK study [2]. These practical and professional challenges appear combined to influence new graduates to achieve financial stability through paid salaried employment where they gain professional experience in a training environment. It is important for countries that support dental education to establish enough training centres to offer new graduates professional training. Where there are insufficient places for graduates, then it may be increasingly incumbent on training institutions to think more widely about opportunities globally in areas where there is a paucity of dentists to meet the needs of the population.

### Long-term career aspirations

The findings suggest that the students in this cohort were clear about their long-term goals. The majority anticipated pursuing the role of a specialist in dentistry with no significant difference by ethnicity or gender overall. Whilst there was a clear preference towards achieving postgraduate qualification, the number of postgraduate programs available in the UAE is limited in both the volume and range of specialities. Of the specialities listed, Orthodontics emerged as the most favoured option followed by Oral and Maxillofacial Surgery. The preference for these two specialties was significantly higher in the ‘Emirati’ ethnic group when compared with ‘Others’; however, given that the findings were based on only one cohort in one dental school in the UAE, they should be treated with caution. Globally, these two specialties are the most common in relation to dentistry [22], and both are associated with higher income [23]. Thus, they provide status and financial benefits which may enhance their attractiveness to students; however, decisions about the level of specialist services should depend on the needs and demands of a population [24], and graduates may need to study abroad to pursue their chosen specialty. A desire to develop extended skills or train as a specialist is not uncommon in contemporary cohorts of students; Gallagher *et al*. [4], reported that over 50% of final year students wished to extend their skills to train as a ‘dentist with special interest’ or a ‘specialist’. Similarly, the studies by Khami *et al.*[25] and Dastjerdi *et al*. [26], demonstrated the high preference for postgraduate qualifications among Iranian dental students; however, even in high income countries with a large established dental profession, only 10-20% of the profession may be specialists [22]. Thus, the findings in the limited literature available, suggest that there may be a high desire amongst present cohorts of students to specialise in dentistry; however, it is increasingly recognised that with global mobility of the dental profession, opportunities to practice as a specialist need not be limited by the needs of the country where educated and trained.

In relation to working patterns, the majority of students reported that they intend to work full-time, which concurs with the findings from the studies of dental schools in the UK [4,27,28]; however, interestingly there was no significant difference between male and female students with regards to working hours which contrasts to UK studies [4,27-30], albeit there was a trend in the same direction. Previous research in the UK has highlighted differences in proposed contributions to the workforce by gender as females perceive that they will bear the responsibility of family and childcare [4,27,31,32]. It important to note that females reported similar influences on their long-term careers as males, except for ‘childcare commitments’ which they were significantly more likely to perceive as important in parallel with their UK counterparts. Given that females are in the majority amongst this student group, their overall contribution to the workforce is important to monitor.

The top three influences affecting the students’ professional career choices were involving, or relating to, good quality of life followed by work related goals. The influence of ‘income and financial security’ and ‘gaining professional experience’ resonates with students’ aspirations for short-term career goals. Along with financial security, the students’ intention to have a good standard of living is again associated with the financial capability of an individual. These findings are similar to those reported by Gallagher *et al*. in the UK where ‘standard of living’ and ‘high income’ and ‘financial security’ are important influences on the career choice of the dental students and newly qualified dentists [4,18].

### Strengths and limitations of the study

This is the first study exploring the views of the final year dental students in the UAE, and the second in a series of papers examining the views of final year dental students’ in the dental school of Ajman University [20]. The study contributes to the literature on final year dental students’ short and long-term career aspirations to select dentistry as a profession as there are limited published studies for this cohort [2,4,33], compared with pre-dental or dental students in other years of their study [17,27,28,34-41]. This research incorporated the views of the dental students from just one dental school in the UAE and hence, care should be taken for relating these findings to the overall UAE population; however, they do raise certain issues for debate and future research.

### Implications and recommendations for future

The study highlights the career aspirations of the dental students and it is important to consider whether these aspirations would affect the provision of oral health care in future. Professional groups are ‘dynamic’, negotiating their status with the state, health systems, and the population whilst influenced by the wider context [42]. These findings highlight the importance of government, academia, and the professional collaborating to ensure that new graduates gain an appropriate induction to the practice of dentistry, whilst also providing for the needs of the population. Ministries of Health have the challenge of creating an adequate healthcare system but also harnessing the skills and expertise of its brightest and best graduates, to retain their services where appropriate and ensure that they can have a sufficiently fulfilling professional career. This can be achieved by examining the oral health needs of the population, career profiles and expectation of dental professionals and taking a public health approach to workforce planning [43]. These findings should be used to inform future workforce needs and particularly for specialist training; however, with global mobility there has to be an acknowledgment that no country is training the dentist workforce for their own country alone. Professional mobility is a fact of life, and thereforeplanning needs to be more sophisticated to take account of workforce that flows into and out of countries [44].

## Conclusion

The career aspirations and future plans of the senior dental students in this dental school revolved around achieving financial, personal and professional goals; however, there may be a tension between professional training opportunities, population health needs and career aspirations. These findings have implications for future that should be considered by the health policy makers in the UAE to harness the potential of this new dental workforce for provision of dental care.

## Competing interests

Dr S Abufanas is the Dean of the dental school in which the study was conducted and Dr H Rashid has contributed to student teaching in the school.

## Authors’ contributions

JG and HR designed the study supported by SA. HR conducted the study facilitated by SA. SG assisted JG with drafting the paper. All authors read and approved the final manuscript.

## Pre-publication history

The pre-publication history for this paper can be accessed here:

http://www.biomedcentral.com/1472-6831/13/39/prepub
